# Faculty and student perceptions of academic counselling services at an academic health science center

**DOI:** 10.1007/s40037-013-0056-1

**Published:** 2013-04-16

**Authors:** Natalie White Gaughf, Penni L. Smith, Dara A. Williams

**Affiliations:** 1 Office of Academic Affairs, University of Mississippi Medical Center, 2500 North State Street, Jackson, MS 39216 USA; 2 Department of Family Medicine, University of Mississippi Medical Center, 2500 North State Street, Jackson, MS 39216 USA

**Keywords:** Academic counselling, Student support, Study skills, Health professional education

## Abstract

There are limited data on support services that facilitate students’ academic success at academic health science centres. The authors explored faculty and students’ perceptions of available academic counselling services (ACS) at an academic health science centre in the Southeastern United States. Participants were surveyed in May and June of 2011 regarding the ACS available at the institution. Fifty-nine percent of faculty respondents (*N* = 471) agreed that academic counselling was a necessary part of the institution, but only 26 % reported knowledge of how to refer students for academic counselling. Only 18 % stated they had previously referred a student for services. Fifty-four percent of student respondents (*N* = 360) agreed that academic counselling was a necessary part of the institution and 60 % stated that they would seek these services if needed. However, only 35 % of students reported that they were aware of how to access the services. These findings suggest a discrepancy between the belief that academic support services have value and their knowledge about how to utilize the services. It is recommended that academic health science centres consider the promotion of available academic support services amongst both faculty and students when designing and implementing programmes to reduce this potential obstacle to service utilization.

## Introduction

Academic health science centres have unique educational considerations that differ from other institutions of higher learning. Specifically, many students enter into this setting unprepared for the rigors of academic study [1]. In addition, some consider the rigors often present in health science education and training a customary part of the overall experience and a good primer for the realities of future professional practice [2]. This logic may result in academic failures [3].

Therefore, the importance of academic support throughout the educational process has been recognized and programmes designed to better prepare students for the rigors of academic coursework in the health sciences introduced [1]. In a study examining students’ utilization of various support services, including academic assistance, researchers found an increase in the overall percentage of students seeking help [4]. Still, further data suggest that the overall numbers of individuals accessing these services may be significantly lower than those evidencing a need [5]. Potential barriers explored and discussed by the current study include faculty and student knowledge and awareness of available academic counselling services (ACS) offered to students within an academic health science centre.

## Method

### Participants

Surveys were distributed to all 963 faculty and 2,067 students at an academic health science centre in the Southeastern United States. The survey was part of a larger needs assessment conducted by the office of ACS. ACS is available to all students on campus and was created to assess problems interfering with academic performance, address the transition to professional school, provide study skills assessment and training, provide time management training, teach testing strategies, assess career goals and interests, address issues related to self-confidence and self-doubt, screen for depression and/or anxiety, provide stress management training, address relationship issues, and/or assist with loss and bereavement concerns. Forty-nine percent of faculty (*N* = 471) and 17 % of students (*N* = 360) completed the survey. The participants represented all professional schools at the institution: Dentistry, Graduate Studies in the Health Sciences, Health Related Professions, Medicine, Nursing, and Pharmacy.

### Instruments

Two surveys were designed to elicit feedback concerning familiarity, access, and perceived value of academic support services offered by ACS. A 5-item survey distributed to faculty consisted of descriptive questions (e.g., school, academic rank), one item containing multiple subparts and utilizing a 5-point Likert scale (strongly disagree, disagree, unsure, agree, strongly agree), and an open-ended question soliciting voluntary comments. A 6-item survey distributed to students consisted of descriptive questions (e.g., school), one item containing multiple subparts and utilizing a 5-point Likert scale (strongly disagree, disagree, unsure, agree, strongly agree), and an open-ended question soliciting voluntary comments. The items with multiple subparts and Likert scale results for both surveys are presented in Table 1.

**Table 1:** Faculty and students’ ratings of academic counselling services

Survey items	Disagree	Unsure	Agree	NA
Faculty survey (*N* = 471)				
I am familiar with the office of ACS*	171(37 %)	91(20 %)	196(42 %)	8(2 %)
I am aware of the services provided by the ACS office	186(40 %)	140(30 %)	133(29 %)	5(1 %)
I know how to refer students to ACS	219(47 %)	113(24 %)	121(26 %)	11(2 %)
I regard ACS as a necessary part of the institution’s student support services	43(9 %)	121(26 %)	271(59 %)	28(6 %)
I have referred students to ACS in the past	292(63 %)	26(6 %)	84(18 %)	58(13 %)
I plan to refer students to ACS in the future	42(9 %)	200(43 %)	181(39 %)	39(8 %)
Student survey (*N* = 360)				
I am familiar with the office of ACS	140(39 %)	62(17 %)	142(39 %)	16(4 %)
I am aware of how to access the office if I choose to utilize ACS services	154(43 %)	71(20 %)	125(35 %)	10(3 %)
I would make an appointment with ACS if I felt the need	38(11 %)	86(24 %)	217(60 %)	18(5 %)
I regard ACS as a necessary part of the institution	30(8 %)	108(30 %)	193(54 %)	29(8 %)
I would recommend ACS to a friend/classmate/colleague	35(10 %)	119(33 %)	156(44 %)	48(13 %)

Omitted responses were not included in the table

* Academic counselling services

### Procedure

The needs assessment surveys were originally distributed by the office of ACS for programmatic improvement. The institutional board determined that the survey effort did not meet the definition of research, as the intent of the effort was limited to needs assessment only. However, this work was carried out in accordance with the declaration of Helsinki and the anonymity of participants was guaranteed. In May 2011, all students enrolled at the institution received an email from each school’s specific student affairs representative with a link to the online survey. In June 2011, all faculty at the institution received an email from the Associate Vice Chancellor of Academic Affairs with a link to the online survey. Both groups received an emailed reminder to complete the survey 7 days after the initial requests. Both surveys were created using Zoomerang online survey software, and survey submission was anonymous. Completion was voluntary, and neither students nor faculty were offered incentives for survey submissions.

## Results

Faculty and students’ Likert ratings were combined to create three distinct categories: disagree (strongly disagree, disagree), unsure, and agree (strongly agree, agree). These can be found in Table 1. Approximately 59 % of faculty agreed that ACS were a necessary component of the institution. However, only 42 % of faculty reported familiarity with ACS, and only 29 % stated that they were aware of the type of services provided. Similarly, 26 % of faculty reported knowledge of how to refer students for academic counselling, while only 18 % stated they had previously referred a student for services. Thirty-nine percent of faculty reported that they planned to refer students for academic counselling in the future.

With regards to perceptions of students, approximately 39 % reported that they were familiar with ACS. In addition, only 35 % of students stated that they understood how to access the services. However, 54 % of students stated that they believed ACS were a necessary part of the institution’s support services, 60 % reported that they would seek ACS if they felt the need, and 44 % reported that they would recommend academic counselling to a friend or classmate.

### Qualitative review of narrative comments

Of the 471 faculty respondents, 57 provided voluntary comments to the prompt ‘Please feel free to provide additional comments about ACS. Of the responses provided, 19 indicated that they would like more information about the services, 13 indicated that they had never heard of the services, three voiced positive statements that the services would be helpful to provide for students, five provided general comments about the procedures for referral to this service, and 16 respondents provided specific feedback about the services or the questionnaire itself.

Of the 360 student respondents, 53 provided comments to the same prompt. Of the responses provided, seven were not included that consisted of statements such as ‘N/A’ and ‘none’. Of the 46 responses remaining, 33 students indicated that they had none or limited knowledge about services offered by the ACS, six indicated that they had never used or did not need the services, four individuals offered solutions for ACS to better advertise on campus, two students reported use or attempted use of services in the past, and one respondent reported a positive view of the services.

## Discussion

The current study is a preliminary examination of the perceptions of faculty and students related to ACS at an academic health science centre. The purpose is to understand how teachers and learners in advanced health education programmes view this form of student support and to assist educators and institutional administrators with implementing programmes aimed at fostering academic success.

The study indicated that although greater than 50 % of faculty and 50 % of students considered academic counselling a necessary component of the institution, far fewer understood how to access the resource. While approximately 40 % of faculty stated that they were familiar with academic counselling and would like to refer students to this resource in the future, substantially less reported prior referral of students (18 %) or knowledge of how to refer a student (26 %). This is troublesome given faculty members’ significant roles in students’ academic development and success within academic health science centres. Supportive faculty interaction has been associated with student success [6], and faculty often serve as mentors for professional development [7]. If faculty have limited knowledge of available academic support services and rarely recommend these services to students, the likelihood that students will receive and benefit from these services may be diminished.

The number of students who regarded ACS as a needed component of the institution was similar to that of faculty. Additionally, 60 % of students stated that they would seek these services if needed. Yet again, similar to faculty reports, fewer students reported current knowledge of how to access these services (35 %). This suggests that while some students are willing to seek help if academic problems arise, their limited knowledge on obtaining the resource may be an obstacle.

## Conclusion

These findings indicate that although faculty and students at an academic health science centre perceived ACS as potentially beneficial, both groups indicated a lack of knowledge with regard to accessing the services. It is suggested that this discrepancy may be reduced via institutional promotion of ACS through greater visibility of the resource at the institution, an easily accessible process for service acquisition, and on-going education on available services and referral processes. Interestingly, descriptive and qualitative survey responses indicated that the survey process led to greater visibility of resources, resulting in a secondary purpose for this survey.

Given faculty’s role in facilitating the academic and professional development of students, it is recommended that faculty be better prepared to ensure that students with academic difficulties are encouraged and directly referred to academic support services. Institutions should consider these suggestions when designing such programmes to ensure that utilization of academic support services is not hindered by a lack of information. The authors hope to stimulate dialogue about support services at academic health science centres through the continued examination of outcomes associated with academic behaviours and interventions.
